# Interaction of Uperin Peptides with Model Membranes: Molecular Dynamics Study

**DOI:** 10.3390/membranes13040370

**Published:** 2023-03-23

**Authors:** Elena A. Ermakova, Rauf Kh. Kurbanov

**Affiliations:** Kazan Institute of Biochemistry and Biophysics, FRC Kazan Scientific Center of RAS, Lobachevsky Str., 2/31, 420111 Kazan, Russia

**Keywords:** uperin, potential of mean force, umbrella sampling, molecular dynamics simulation

## Abstract

The interaction of antimicrobial and amyloid peptides with cell membranes is a critical step in their activities. Peptides of the uperin family obtained from the skin secretion of Australian amphibians demonstrate antimicrobial and amyloidogenic properties. All-atomic molecular dynamics and an umbrella sampling approach were used to study the interaction of uperins with model bacterial membrane. Two stable configurations of peptides were found. In the bound state, the peptides in helical form were located right under the head group region in parallel orientation with respect to the bilayer surface. Stable transmembrane configuration was observed for wild-type uperin and its alanine mutant in both alpha-helical and extended unstructured forms. The potential of mean force characterized the process of peptide binding from water to the lipid bilayer and its insertion into the membrane, and revealed that the transition of uperins from the bound state to the transmembrane position was accompanied by the rotation of peptides and passes through the energy barrier of 4–5 kcal/mol. Uperins have a weak effect on membrane properties.

## 1. Introduction

Antimicrobial peptides (AMPs) constitute the main component of the innate defense system of all organisms, including humans, plants, insects, amphibians and others. AMPs demonstrate a wide range of activities, including fungicidal, antibiotic, antiviral, wound-healing, antidiabetic, analgesic, and anticancer properties [1,2,3,4].

Some AMPs demonstrate activity against several types of pathogens, but others exhibit highly specific activity. For example, magainin, melittin, δ-lysine bind to both zwitterionic and anionic membranes, defensins bind anionic and do not interact with zwitterionic membranes [5,6].

The development of new antimicrobial peptides as therapeutic agents requires an understanding of their structure and mechanisms of action. Investigation of peptides with antimicrobial properties is also necessary due to the increasing resistance of pathogens against available drugs.

The molecular mechanism of peptide activity is a complicated multistage process that includes peptide–membrane interactions as a first and significant step. Some peptides are able to cross cell membranes without their disruption and target intracellular components (e.g., buforin). Other AMPs kill pathogens by disrupting their membranes (e.g., magainin, aurein). Several membrane-related mechanisms have been developed, including the carpet mechanism and the pore-forming model [7,8,9,10]. In the carpet model, AMPs bind to the surface of membranes, cover it in a carpet-like manner, and cause membrane disruption [7]. In the pore formation model, the AMPs form barrel or toroidal pores stabilized by peptides (melittin, protegrin, magainin) [11,12,13].

The specific mechanism of the peptide–membrane interaction depends on the peptide amino acid sequence, its size, shape, and charge, as well as the lipid composition of the membrane and environmental factors [11,12,13].

AMPs exhibit high variability in sequence and structure. They can have alpha/beta structures (defensins), beta-rich (protegrins, megin) or alpha-helical structures (melittin). Some AMPs, for example, magainin, aurein, maculatin, and caerin, are unstructured in aqueous solution and adopt amphipathic α-helical structures in the presence of membranes or organic solvent mixtures [11,12,13].

Various simulation strategies and, especially, molecular dynamics simulations are commonly used to access the peptide–membrane interactions, to analyze the peptide binding and accumulation on the membrane, the penetration of peptides through membrane or formation of transmembrane pores, in order to study the influence of the peptides on the membrane structure and integrity [14,15,16,17,18,19].

Here, we applied MD simulations to study the interaction of new peptides from the uperin family with a model bacterial plasma membrane. Uperins are 17–18 residues in length and carry a small positive charge. They can exist in different structural forms depending on environmental factors. Uperin can form amphipathic α-helices in organic solvent mixtures and membranes, beta-rich amyloid structures in salty solutions, and unstructured random coil structures in water [20].

Uperins are extracted from the skin of Australian toadlets and, like other peptides from amphibians, demonstrate antimicrobial properties [21]. Another interesting feature of uperins is that they demonstrate amyloidogenic properties; they can form fibrils with a structure similar to those formed by Aβ-peptides [22]. Calabrese et al. also showed that uperin’s fibrils are cytotoxic to model neuronal cells similar to proteins implicated in neurodegenerative diseases [22]. Recently, it was shown that uperins can form beta-sheet-rich amyloids and alpha-helical cross amyloids as well [20].

Here, we investigated the interaction of two uperin peptides (wild uperin Up3.5 and its mutant having the alanine residues in the seventh position instead of arginine) with a model bacterial membrane consisting of two types of lipids: 1-palmitoyl-2-oleoyl-sn-glycero-3-phosphoethanolamine (POPE) and 1-palmitoyl-2-oleoyl-sn-glycero-3-phosphoglycerol (POPG). Binding of peptides to the lipid membrane was studied using conventional molecular dynamics (MD). To investigate the possibility of peptide insertion into membranes, we performed a series of umbrella simulations and computed the potential of mean force (PMF) profile associated with the insertion of a single uperin molecule into a bilayer. An influence of peptide structure on the PMF and the preferred location and orientation of molecules in the bilayer, as well as the effect of the peptides on the membrane structure and integrity, was analyzed.

## 2. Methods

Uperin 3.5 is a short antimicrobial peptide consisting of 17 amino acids (GVGDLIRKAVSVIKNIV). Modeling was performed for wild uperin (Up) and its mutant (Up7a), which contains alanine residue in the seventh position instead of Arg7. The structure of wild-type Up in the α-helical form was obtained from the Protein Data Bank (code 6FLT) [20]. Uperin mutant was constructed using the VMD program [23]. Consistent with the experimental conditions, wild-type uperin and its mutant were amidated at the C-terminus. Modeling was performed for two conformations of peptides: peptides in alpha-helical form (UpH, Up7aH) and in extended unstructured form (UpE, Up7aE).

All systems were prepared using the CHARMM-GUI (https://www.charmm-gui.org/, accessed on 5 February 2023) webserver [24]. A model membrane composed of 1-palmitoyl-2-oleoyl-sn-glycero-3-phosphoethanolamine (POPE) and 1-palmitoyl-2-oleoyl-sn-glycero-3-phosphoglycerol (POPG) was built as a bilayer with a 3:1 molar mixture of POPE and POPG lipids. Each leaflet of the bilayer contains 100 lipids. Membrane was included in the water box and corresponding numbers of counter-ions were added to neutralize the system at an ionic strength of 150 mM.

Two types of calculations were carried out. In the first type, several unbiased simulations were performed. Each system was modeled as minimum 500 ns. Details of the simulations are provided in Table 1. In order to be able to evaluate the effect of peptides on the properties of the membrane, additional modeling of the membrane without peptide molecules was carried out.

In the second type of simulation, an umbrella approach was used to obtain the potential of mean force [25]. The influence of modeling parameters, and in particular the influence of the initial position of molecules on PMF, was considered in detail [14,26,27].

Initially, peptides were placed in the center of the membrane perpendicular to its surface, with the C-terminus of peptides pointing toward the bottom leaflet. The pull code supplied with the GROMACS suite (https://www.gromacs.org/, accessed on 5 February 2023) [28] was used to obtain starting configurations for subsequent umbrella sampling (US) calculations. Peptide molecules were pulled along the reaction coordinate (the distance between the centers of mass of peptide and a lipid bilayer in the direction perpendicular to the bilayer surface) at a velocity of 0.00002 nm/ps and a force constant of 1000 kJ∙mol^−1^·nm^−2^. The orientation and conformation of the peptides were not constrained. Head groups of lipids were restricted with a force constant of 100,000 kJ∙mol^−1^·nm^−2^.

Snapshots obtained from the pulling simulation trajectories were used to generate the starting configurations for each window of the umbrella sampling simulation (from 0 to 4.4 nm along the reaction coordinate with a spacing of 0.1 nm between the windows). For each system, a series of 45 separate simulations of 50 ns, each with a force constant of 1000 kJ∙mol^−1^·nm^−2^, were performed, in which the peptide was constrained to a given depth in the bilayer by a harmonic restraint on the z-coordinate. The last 30 ns of the trajectories were used for the generation of PMF. The orientation and conformation of the peptide were not restrained.

The Weighted Histogram Analysis Method (WHAM), implemented in the GROMACS package, was used to extract the PMF and calculate the change in free energy (ΔG) as a function of the distance between the peptide and bilayer centers along the z-axis normal to the plane of the bilayer.

All simulations were performed with the GROMACS 21 MD package [29]. A CHARMM36 (https://www.charmm.org/archive/charmm/resources/charmm-force-fields/, accessed on 5 February 2023) force field was used for all components of the system [30]. Non-bonded interactions were calculated within a cutoff of 1.2 nm for Lennard-Jones and electrostatic interactions. Periodic boundary conditions (PBC) were employed for all simulations, and the particle mesh Ewald (PME) method was used for long-range electrostatic interactions. The temperature was maintained at 303 K, a Nosé-Hoover temperature coupling method with a time constant of 1 ps was used, and for pressure coupling, a semi-isotropic Parrinello−Rahman method with a time constant of 5 ps and a compressibility of 4.5 × 10^−5^ bar^−1^ was used. The pressure was maintained at 1 bar. The simulation time step was set to 2 fs. Each model was equilibrated using the typical six-step CHARMM-GUI protocol for 2 ns [24].

The GROMACS and VMD (https://www.ks.uiuc.edu/Research/vmd/, accessed on 5 February 2023) tools were used for the analysis of the bilayer characteristics. To analyze the influence of peptide on the membrane structure, the order parameters were calculated:

S_CH_ = 0.5⟨3cos^2^θ−1⟩ (1)

where θ is the angle between the C−H bond vector and the bilayer normal. S_CH_ was defined for every methylene/methyl group in the chains. The angular brackets denote time and ensemble averaging.

The orientation of peptides was characterized by the angle between the main principal axis of inertia (corresponding to the smallest eigenvalue of the moment inertia tensor) and the normal to the bilayer and was defined using the g_principal function in GROMACS. For the calculation of the number of water molecules penetrating the bilayer, our own code was written. For visualization of molecular structures, the VMD program was used [23].

## 3. Results

**Figure 1:**
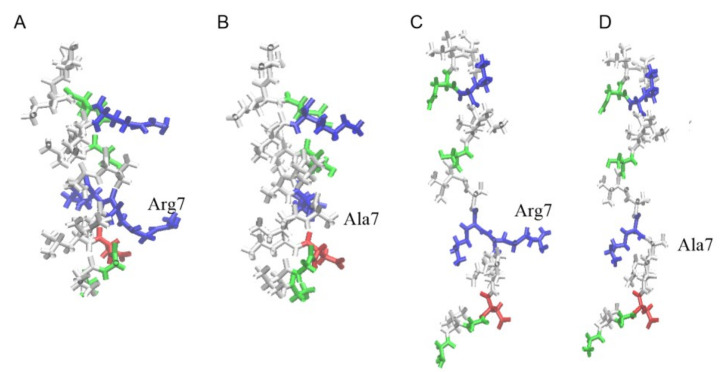
Structure of uperin and mutant in alpha-helical form (**A**,**B**) and in extended configuration (**C**,**D**). Peptides are shown in bonds and are colored according to residue types.

Structures of uperin peptides are shown in Figure 1. The peptides adopt an amphipathic α-helical structure, where hydrophobic residues form a large hydrophobic site on one side of the helix and polar residues are located on the other side. When uperin is in an extended configuration, no significant hydrophobic and hydrophilic zones are formed (Figure 1). Up has a total positive charge of +3, and the main part of the positively charged residues are closer to the N-terminus (Gly1, Arg7, Lys8), while the C-terminus of uperin is amidated, and the total charge of peptide is slightly shifted to the N-terminus. Up7a has a total charge of +2 due to replacement of the Arg7 residue with alanine.

### 3.1. Unbiased MD Simulations

To investigate the interaction of uperins with the model membrane and to detect stable configurations of peptides relative to the membrane, we conducted several MD simulations by placing the peptides approximately 3–4 nm away from the bilayer center in different orientations relative to the membrane surface (Table 1).

Overall, we performed unbiased simulations for wild uperin Up and its mutant Up7a in alpha-helical form (UpH and Up7aH) and in unstructured random coil form (UpE and Upa7E), with two different initial positions of peptides relative to the bilayer.

#### 3.1.1. Unbiased MD Simulations of Uperins in Alpha-Helical Form

Initially, peptides in alpha-helical form were placed in the solution far away from the bilayer surface in an arbitrary orientation relative to the membrane.

**Figure 2:**
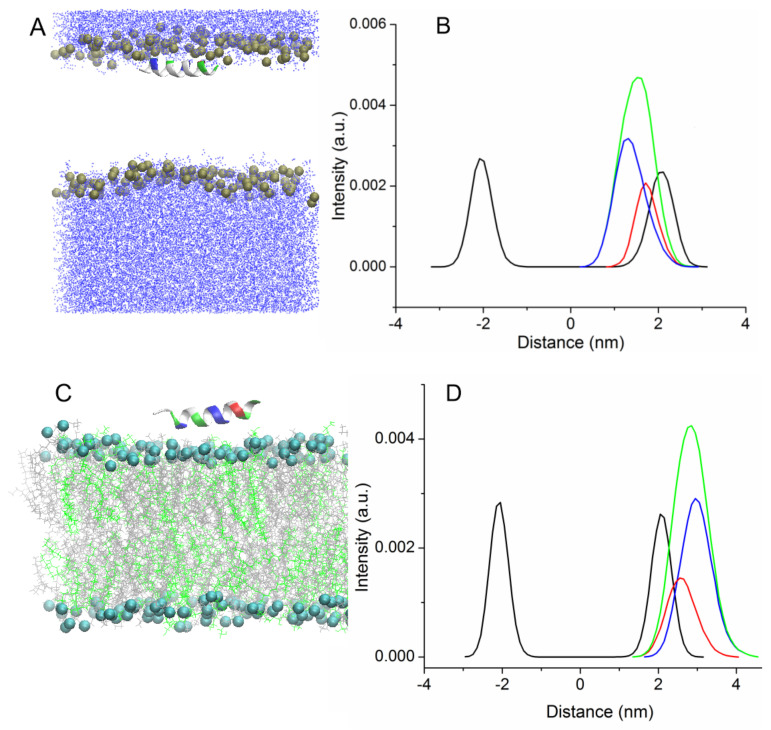
Snapshots of the systems for the simulations Tr1 (**A**) and Tr3 (**C**). Peptide is shown in the cartoons and is colored according to residue types. Density profiles (**B**,**D**) along the bilayer normal are plotted for the phosphate group (black), peptides (green), hydrophilic residues (red), and hydrophobic residues (blue). In Figure 2A, lipids are hidden for clarity. In Figure 2C, water molecules are not shown.

In one case, both peptides were quickly inserted into the membrane for the first 100–150 ns (trajectories Tr1 and Tr2). Figure S1 shows the time evolution of the angle between the main principal axis of UpH peptide and the normal to the membrane surface. The peptide rotated several times around its axis, and was then inserted into the membrane parallel to its surface, and this orientation remained stable until the end of the simulation. The process of embedding the peptides is accompanied by a distortion of their secondary structure, which quickly recovers after passing through the zone of the head groups (Figure 2A). 

No helical kink or bending of the peptide was observed for wild-type uperin and its mutant. The peptides were located near the lipid head group during the remainder of the simulation, while the distance between the centers of the peptide and the bilayer was about 1.3 nm and 1.7 nm for Upa7H and for Up peptides, respectively. The hydrophobic face of the peptide points toward the bilayer core, and interaction of charged residues with lipid head groups stabilizes the peptide position. Figure 2A shows the final configuration of Up peptide and the density profiles for lipid phosphorus atoms and peptides averaged over trajectories along the direction perpendicular to the bilayer surface. Figure 2B illustrates the difference in the penetration of hydrophobic and hydrophilic residues into the membrane. The hydrophobic residues are found deeper inside the bilayer than the hydrophilic residues.

During the simulation, no translocation of the peptides into the solution or further penetration of the peptides to the center of the bilayer was detected. This position is typical for amphipathic peptides. It has been shown in several studies that amphipathic peptides insert themselves into the bilayer under the lipid head groups [14,31,32].

In the other case, uperin molecules were absorbed on the bilayer surface (trajectories Tr3 and Tr4). Peptides also associate with the membrane surface, but they are deposited on the surface of the membrane with the hydrophobic face of the peptide pointing toward water and polar residues interacting with lipid head groups. Although the calculation of interaction energy between the peptides and bilayer reveals that the position is not energy favorable, peptides stayed in this position for about 150–200 ns, and the distance between the centers of peptides and the membrane was equal to 2.8–3 nm (Figure 2C). As shown in the density plot (Figure 2D), the charged residues are closer to the membrane center than the hydrophobic residues. Afterward, Up7aH rotated around the helix axis and, reoriented by the hydrophobic side, faced the lipid head groups and was inserted into the bilayer. The peptide rotated in such a way that the C terminus first penetrated into the hydrophobic region of the membrane. For the UpH peptide, two variants were observed. In one case, similar to the Up7aH peptide, UpH rotated and embedded itself into the bilayer. In another case, UpH began to change its configuration, lost its alpha-helical structure, and unwound into a random coil. This took about 600 ns.

**Figure 3:**
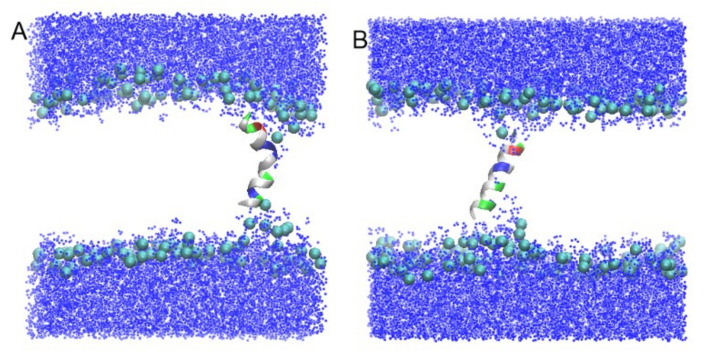
Snapshots of the systems for the simulations Tr5 (**A**) and Tr6 (**B**). Lipid tails are hidden for clarity.

Two additional MD simulations of uperins in alpha-helical form were performed for peptides inserted inside the membrane in the transmembrane orientation (Tr5 and Tr6) (Figure 3). 

Both peptides are stable in the transmembrane orientation until the end of the simulations. However, there is a difference in their favorable orientations. Wild-type uperin is almost perpendicular to the membrane surface, but it changes its form and adopts a “U-shape” form (Tr5, Figure 3A). To characterize the peptide bending, the angle formed by the C-alpha atoms of residues 2, 9, and 16 was calculated. For wild-type uperin, this angle was equal to 150° ± 12°. Polar residues are located on the concave side of the peptide, and hydrophobic residues are on the convex side to reduce the unfavorable interaction of the charged residues with the membrane hydrophobic region. The C- and N-termini are anchored to the polar head group region. Positively charged residues are closer to the N-terminus of the peptide, and the center of mass (COM) of peptide is slightly shifted to the upper leaflet of the bilayer and is placed about 0.3 nm from the center of the bilayer when the peptide is in a stable transmembrane state.

Up7aH, having more hydrophobic residues, did not change its form, and the angle between the C-alpha atoms of residues 2, 9, and 16 was 170° ± 10°. It is placed at the center of the membrane, but it deviates from the vertical position and the angle between the helix axis and the membrane normal is about 20° (Tr6, Figure 3B). This position is characterized as a pseudo-transmembrane state. It was observed for interaction of melittin with the POPC lipid bilayer [14].

In general, two stable states were detected for uperin molecules in alpha-helical form. One of them is a transmembrane state, and another is a bound state (B state). No spontaneous translocation of peptides from one stable state to another was observed during the simulations.

#### 3.1.2. Peptides in Random Coil Extended Configuration

**Figure 4:**
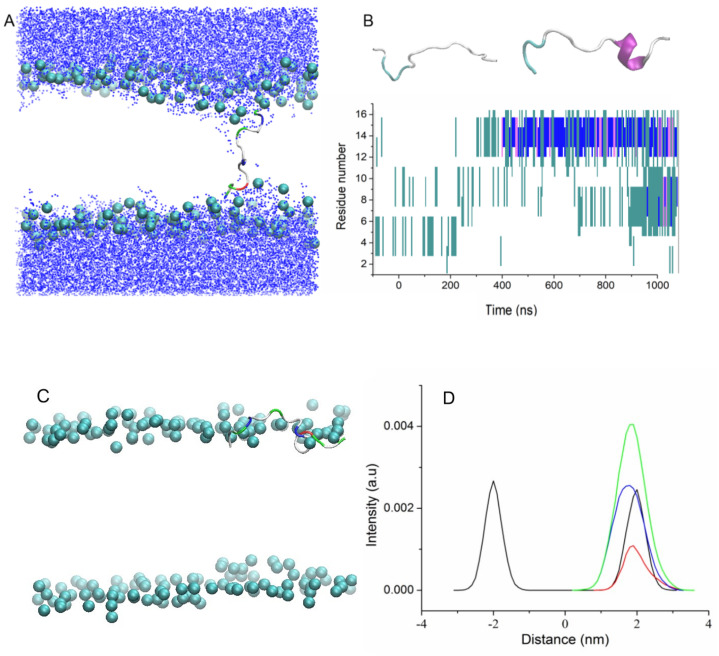
Snapshots of the systems for the simulations Tr7 (**A**) and Tr9 (**C**). Peptides are shown in the cartoon and are colored according to residue types. (**B**) Time evolution of the secondary structure of peptide. White, cyan, blue, and purple colors correspond to a random coil, a turn, a π-helix, and an alpha-helix, respectively. Density profiles (**D**) along the bilayer normal are plotted for the phosphate group (black), peptides (green), hydrophilic residues (red), and hydrophobic residues (blue). In Figure 4A,C, lipids are hidden for clarity.

Two types of MD simulations were also performed for the peptides in random coil unstructured conformation (Figure 4).

In the first type of simulation, peptides UpE and Up7aE were inserted vertically in the bilayer, with the N-terminus of the peptide located near the upper leaflet of the bilayer and the C-terminus of the peptide pointed toward the bottom leaflet (trajectories Tr7 and Tr8).

During MD simulations, both peptides were stable inside the membrane in a tilted orientation across the bilayer with an averaged angle between the main principal axis and the membrane normal of 15° for Up7aE and 18° for UpE peptides, and the N- and C-termini were extended to both leaflets (a snapshot of the trajectory Tr7 is shown in Figure 4A, as an example).

It is often assumed that AMPs, which are unstructured in water solution, adopt a helical conformation when they are immersed in the hydrophobic bilayer core. During the simulation, partial folding of peptides from random coil conformation into alpha-helical structure was observed. Uperins begin to fold at 400–700 ns. Folding of peptides begins from the N-terminus and involves 15–20% of residues after 1 μs of simulation. The partially folded part of the peptides did not demonstrate any attempt to unfold. To further characterize the folding of peptide into the membrane, we extended one of the trajectories to 2 μs. Folding, which began at the N-terminus, gradually involved neighboring residues, and 40% of the residues were involved in alpha-helical structure in 1.5 μs.

In the second type of simulation, similar to trajectories Tr1 and Tr2, UpE and Up7aE peptides were placed in the solution far away from the bilayer surface in random orientation relative to the membrane surface (Tr9 and Tr10). Both peptides quickly associate with the membrane surface but do not enter the zone of carboxyl groups; they are mainly located near the phosphorus atoms. The distance between the centers of the peptide and the bilayer is about 1.9 nm and 2.2 nm for Up7aE and UpE peptides, respectively. Up7a peptide, having more hydrophobic residues than wild-type uperin, is inserted deeper into the bilayer and is located near the phosphorous atoms. Figure 4C shows the density plots for Up7aE peptides in extended configuration. Hydrophobic and hydrophilic residues are equally embedded in the membrane.

The transformation of the peptide structure from a random coil into an alpha-helical structure was also detected for peptides adsorbed to the membrane surface. It starts from about 300–400 ns from the N-terminus residues and reaches about 20–25% after about 1μs. The time evolution of the secondary structure of peptide is shown in Figure 4B.

Therefore, unbiased MD simulations of wild-type uperin and its mutant reveal that, regardless of the structural form, both peptides have two stable configurations relative to the membrane: a bound state and a transmembrane state. In the T state, structured and unstructured forms of peptides are located inside the membrane in a vertical or slightly tilted orientation and form stable pores. In the bound state, peptides bind to the membrane parallel to the membrane surface. Depth of protein insertion depends on both the peptide structure and on the balance of hydrophobic and hydrophilic residues of peptides. Peptides with a large positive charge are located further from the membrane center. The folding of peptide into an alpha helix promotes the formation of an extended hydrophobic zone and the incorporation of the peptide into the region of carboxyl groups.

### 3.2. Umbrella Simulation of Peptides in Helical Conformations

To more deeply analyze the process of uperin binding from water to the lipid bilayer and its insertion into the membrane and to compare the stability of different states, we performed a series of biased MD simulations using the umbrella sampling technique.

The calculation of the free energy profile requires very extensive sampling of all possible conformations and is a very time-consuming procedure [26,27]. We deem that the slow rate of pulling the peptide from the center of the membrane and the relatively small force constant allow the peptide to reorient and obtain the most energetically favorable orientation during the pulling process, and this would therefore correspond to a more energy-favorable path. 

We started our pulling simulations from a conformation obtained in a regular MD simulation (Tr5 and Tr6). Initially, the peptides in helical conformation were placed at the center of the membrane in a vertical, transmembrane orientation. The N-terminus was closer to the upper leaflet of the bilayer, because MD simulation revealed that the self-penetration of peptides began at the C-terminus. Peptide molecules were pulled along the reaction coordinate from the center to the outside of the membrane. During the pulling process, the peptides rotate from a vertical orientation to an almost horizontal state. At a distance of 1.5–1.7 nm from the membrane center, the peptides are located in the region of the carbonyl groups near the head group region of the bilayer in an almost parallel orientation with respect to the bilayer surface. When the peptides are pulled further into the water solution, they are reoriented perpendicular to the membrane surface, with the N-terminus pointed to the membrane. Although the rotation of peptides is not restricted, they begin to rotate freely only away from the membrane surface, where interaction with the membrane becomes insignificant.

**Figure 5:**
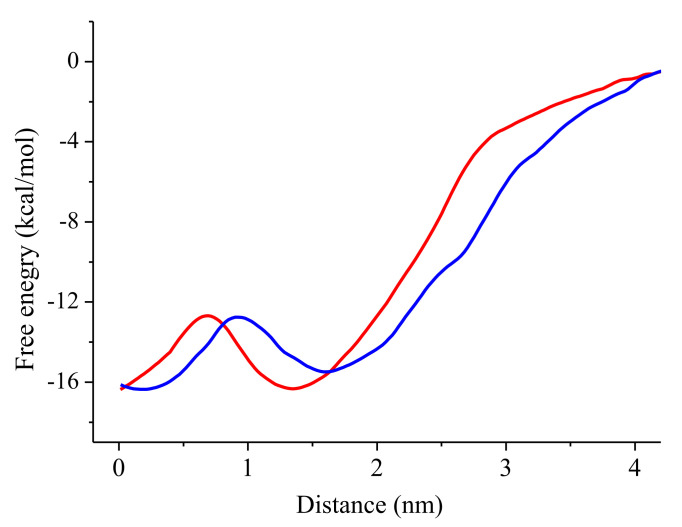
PMF for the insertion of wild uperin (blue line) and its alanine mutant (red line) into the POPG/POPE bilayer.

The peptide coordinates obtained from the pulling simulation trajectory were used as initial positions for the umbrella simulations. Figure 5 displays the change in free energy depending on the distance between the centers of mass of the peptide and the bilayer. The PMF curves were aligned so that the peptide’s relative free energy in bulk water corresponded to zero. The shape of the free energy profiles was similar for both peptides.

The calculated PMF plots are typical for amphipathic peptides. Similar profiles were obtained for the insertion of other AMPs, such as melittin and δ-lysin, into a POPC bilayer [5,14].

When peptides approach the bilayer from the solution, free energy monotonically decreases, confirming the leading role of electrostatic interactions and showing that binding of peptides is energetically favorable. The PMFs have two minima, one at the center of the bilayer and another at the bilayer-water interfacial region, which correspond to two stable states obtained by MD. For UpH peptide, this minimum is located at 1.45–1.7 nm from the membrane center and at 0.4–0.3 nm from the phosphorus atoms. For the more hydrophobic peptide Up7aH, the minimum is slightly shifted to the membrane center and is located at a distance of 0.5–0.7 nm from the lipid head groups.

The introduction of the peptide deeper into the bilayer is accompanied by an increase in free energy due to a decrease in favorable electrostatic interaction with the lipid head groups and an increase in the unfavorable interaction of charged residues with the hydrophobic core. The insertion of peptides into the bilayer and a steric resistance of lipids drive them to rotate and form a vertical, transmembrane orientation. When peptides reach the membrane center, the interaction of peptides with both bilayer leaflets compensates for the unfavorable interaction of charged residues with the hydrophobic core.

The second minimum in the PMF profile corresponds to the transmembrane position of peptides. The second minimum for Up7aH peptides coincides with the center of the membrane, while for the wild-type uperin, it is shifted by 0.2–0.3 nm due to the stronger interaction of the positively charged N-terminus with the anionic atoms of the leaflet than the interaction of the amidated C-terminus. Both minima are almost equivalent; the free energy difference between the transmembrane and the interfacial configurations is very small, about 1 kcal/mol. For δ-lysin, the transmembrane configuration corresponds to the global minimum in the PMF, although the free energy difference between the transmembrane and the interfacial configurations is very small, about 2 kcal/mol. For melittin into a POPC bilayer, the transmembrane orientation is more stable than the surface bound state by roughly 4 kcal/mol [5,14]. For many other AMPs, the transmembrane configurations are less favorable than the membrane-bound state.

Between the two minima, there is a barrier of 4–5 kcal/mol. To overcome the barrier and move deeper into the bilayer, the peptides have to rotate from a horizontal to a vertical orientation. From the horizontal position at the first minimum, the peptide reorients in such a way that the less charged C-terminus moves from the upper leaflet to the center of the bilayer.

The free energy difference between the molecule In the unbound and bound states is −15.5 kcal/mol for wild-type uperin and −16.5 kcal/mol for the mutant. Similar values for binding energy were reported for other AMPs [11,12,13,33]. Nangia et al. investigated membrane binding and folding properties of membrane lytic peptide of Flock House virus. They observed ΔG of binding values of −15.0 kcal/mol, −22.5 kcal/mol, −18.5 kcal/mol for γ1 peptide in helical form with POPC, POPG, and POPC/POPG bilayer, respectively. When peptides were in unstructured form, ΔG of binding was equal to −1.5 kcal/mol, −11.0 kcal/mol, −7.5 kcal/mol for the interactions of peptides with POPC, POPG, and mixed bilayers, respectively [33].

We calculated the change in free energy of peptide adsorption by integrating the PMF along the reaction coordinate (from 1.7 nm for Up and 1.5 nm for Up7a peptides, where PMFs show minimum, to 4.2 nm, where free energy is equal to 0) and obtained a value of −18.4 kcal/mol for wild uperin and −17 kcal/mol for Up7a.

Although the energy barrier between the two stable states (B and T) is about 4–5 kcal/mol, we did not observe spontaneous movement of peptides from one stable state to another for regular MD simulations.

### 3.3. Influence of Peptides on Membrane Properties

The interaction of the peptide with the membrane changes the membrane structure and induces an alteration of the physical-chemical properties of the membrane. To characterize the influence of peptides on the membrane properties and to make it possible to compare the calculated results with those in other publications, we calculated several parameters: the thickness of the bilayer, the area per lipid, the lipid order parameters, the average number of lipid head groups inside the pore, the average number of water particles in the lipid bilayer, and the permeation rate of water molecules through the membrane.

Insertion of peptides into membrane induces the deformation of the lipid bilayer structure that is typical for amphiphilic peptides. When uperins are in the T state, they induce a small deformation of the lipid bilayer structure. The positively charged N-terminus and Arg7, Lys 8, and Lys14 residues interact with the head group atoms of the lower and upper leaflets, causing their bending (Figure 3A,B). The membrane head groups reorient inside the membrane, creating semitoroidal pores in the bilayer. Semitoroidal pores have previously been observed for peptides with alpha-helix structures and beta-structures [10,34]. In the semitoroidal configuration, lipid molecules bend into the bilayer but do not form a continuous leaflet [10]. In combined membranes consisting of two types of lipids, POPE and POPG, the peptide is surrounded mainly by anionic POPG lipids. Peptides Up and Up7a in alpha-helical form induce similar distortions of the bilayer. Phosphorus atoms form a funnel, and four to eight lipid head groups are located within 1–1.5 nm from the membrane center. The minimum distance between the phosphorus atoms of the opposite leaflets reduces to 1 nm for wild-type uperin and 1.3 nm for Upa7H. The peptides in unstructured extended form in transmembrane position have less effect on the membrane structure (Figure 4A). The minimum distances between the phosphorus atoms in the upper and lower leaflets were equal to 1.7 nm and 1.8 nm for UpE and Up7aE, respectively.

**Figure 6:**
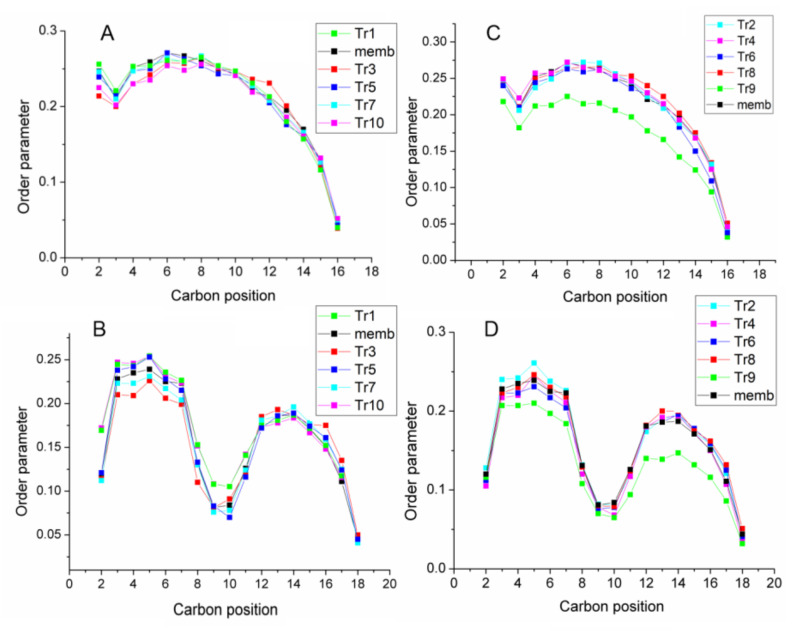
Profile of the uperin effects on the order parameters S_CH_ of POPG lipids for chains sn1 (**A**), sn2 (**B**) of wild-type uperin and for chains sn1 (**C**), sn2 (**D**) of mutant peptide. For comparison, profiles for membrane without peptides are shown in black.

To characterize the effect of peptides on the membrane properties, we calculated the lipid order parameter S_CH_ along the lipid acyl chains for POPE and POPG lipids. Order parameters are a measure of orientation mobility of the lipid hydrocarbon segments along the acyl tails. The order parameters were calculated separately for the palmitoyl (sn1) and oleoyl (sn2) chains of the lipids. Figure 6 shows order parameters averaged over the last 100 ns of the trajectories. Lipids in contact with uperins have more disordered acyl chains than the lipids placed away from the peptides. However, on average, we found no significant effect of peptides on the S_CH_ profiles for most of the systems studied. Surprisingly, the Upa7E peptide in an unstructured form has a significant effect on lipid motility. 

Figure 4C shows that the Upa7E peptide is located directly in the zone of phosphorous atoms, increases the area per lipid, and decreases the thickness of the lipid bilayer (Table S1), while the Up peptide is located a little further away and has no effect on the lipid ordering.

Insertion of peptides into the membrane facilitates the penetration of water molecules through the membrane. The diffusion of water and ion molecules from one side to the other of the bilayer was evaluated. The insertion of peptides increases the number of water particles present in the bilayer and crossing the bilayer. On average, 11–12 water particles were present in the bilayer region during the simulations, while two water molecules were detected in the simulation of the bilayer without peptides. The rate of penetration of water molecules was calculated as the number of molecules crossing the bilayer center per 1 ns. As expected, the maximum rate was detected for pores formed by peptides in helical form, which was equal to 0.2 particle/ns. Peptides in the bound state slightly increase the penetration rate of water molecules compared to the membrane without peptides. In this case, the penetration rate equals 0.04–0.06 particle/ns; for pure membrane this value is 0.02 particle/ns (Table S1). The insertion of chloride ions into the bilayer was not detected in our simulations. The minimum distance between the ions and the bilayer center reached 1 nm.

Comparison of the calculated results with those obtained for other amphiphilic antimicrobial peptides reveals that uperin peptides are inserted into membranes similarly to other antimicrobial peptides but have less deformation effect on them. A small net charge and extended configuration of peptides allow them to insert into the membrane without its distortion.

## 4. Discussion

Peptides exhibiting antimicrobial activity are very diverse in terms of amino acid sequence, spatial structure, size, and net charge, as well as with respect to the balance of hydrophobic and hydrophilic residues that defines the mechanisms of their interaction with cell membranes. Several models have been developed to describe the mechanisms of binding and permeation of molecules through biological membranes [7,8,9,10,11].

In this work, we performed molecular dynamics simulations to study the binding of uperin peptides to the model bacterial plasma membrane consisting of two types of lipids, POPE and POPG. Uperins, short cationic peptides, are unstructured in aqueous solution and adopt amphipathic α-helical structures in the presence of membranes [21]. The influence of two structure forms (alpha-helical and extended unstructured form) of wild-type uperin and its mutant on the peptide–membrane interaction was analyzed.

Two stable configurations of peptides were found. Peptides can be bound to the membrane surface or inserted into the membrane in the transmembrane state. In the bound state, the peptides in helical form are located right under the head group region. Wild-type uperin and its mutant are in parallel orientation with respect to the bilayer surface and maintain high helicity content throughout the simulation. This is in agreement with results for other AMPs. It has been shown in numerous simulation studies [11] that amphipathic peptides insert themselves into the bilayer and form a stable helical conformation right under the head group region of the bilayer. Peptides in unstructured form bind to the membrane surface but do not enter the zone of carboxyl groups; they are mainly located near the phosphorus atoms. The depth of protein insertion depends both on the peptide structure and on the balance of hydrophobic-hydrophilic residues of peptides. Peptides with a large positive charge are located further from the membrane center. The mutant with more hydrophobic residues is located deeper into the center of the bilayer. Peptides in unstructured form inserted into the membrane or located on its surface begin to fold into π-helix and then into the alpha-helix. This is in agreement with the experimental observation that uperin peptides are in helical form in membranes and organic solutions [20].

The second stable position of uperin peptides is the transmembrane state. Wild-type uperin adopted a slightly bending conformation, while positions of mutant in helical form and both peptides in unstructured form are characterized by pseudo-transmembrane positions: peptides are in tilted orientation relative to the membrane normal. In all cases, uperins interact with both leaflets, stabilizing their position.

The potential of mean force was calculated to characterize the process of binding the peptide from water to the lipid bilayer and its insertion into the membrane and to compare the stability of different states.

Many studies have shown how small differences in structure result in differences in the shape of the PMF and the preferred location and orientation of the molecules [11,35,36,37]. MacCallum et al. calculated the distribution of small molecules mimicking 17 natural amino acids in a lipid bilayer and revealed that the shape of PMF depends on the size, shape, polarity, charge, and hydrophobicity of the molecule [35]. Gkeka and Sarkisov classified alpha-helical peptides into several groups based on the distribution of hydrophobic and hydrophilic residues along the helix, calculated the PMF for peptide translocation across the lipid bilayer, and demonstrated that each class has a distinct shape of PMF [36].

Umbrella sampling simulations reveal that the transition of uperins from the bound state to the transmembrane position is accompanied by the rotation of peptides and passes through the energy barrier of 4–5 kcal/mol. The profile of PMF in Figure 5 resembles those obtained for other antimicrobial peptides [5,14,36,37]. The main difference is in the value of the free energy barrier. Obtained results are significantly smaller than the corresponding values for other AMPs. For example, the introduction of the antimicrobial peptide melittin into the POPS bilayer passes through the barrier between the surface-bound and transmembrane states of 13.2 kcal/mol [14], similar barrier for δ-lysin in the POPC bilayer equals 10.2 kcal/mol [5]. The barrier of 8 kT needs to be overcome by LS3 peptide to cross the membrane–water interface and adopt a transmembrane orientation [36].

Another interesting feature of uperins is the weak effect of peptides on membrane structure. Comparison of the calculated results with those obtained for other amphiphilic antimicrobial peptides reveals that uperin peptides are inserted into membrane similarly to other antimicrobial peptides, but have a smaller deformation effect on it. The small net charge and extended configuration of peptides allow them to be inserted into the membrane without its distortion.

## 5. Conclusions

Here, we applied MD simulations to study the interaction of uperin peptides with a model bacterial plasma membrane. Uperins are 17 residues in length and carry a small positive charge. Wild-type uperin and its alanine mutant in both alpha-helical and extended unstructured forms demonstrate two stable states relative to the membrane: bound and transmembrane states. The potential of mean force was calculated for uperins and characterized the process of peptide binding from water to the lipid bilayer and its insertion into the membrane. PMF reveals that the alanine mutant binds to the membrane slightly stronger than wild-type uperin. The transition of uperins from the bound state to the transmembrane position is accompanied by the rotation of peptides and passes through the energy barrier of 4–5 kcal/mol. Uperins have a weak effect on the membrane properties.

## Figures and Tables

**Table 1:** Simulation parameters.

Trajectory Number	Peptide	Initial State	Final State	Conformation	Length of Trajectory (ns)
Tr1	Up	outside	B	Alpha helix	600
Tr2	Up7a	outside	B	Alpha helix	500
Tr3	Up	outside	B	Alpha helix	1100
Tr4	Up7a	outside	B	Alpha helix	500
Tr5	Up	inside	T	Alpha helix	1400
Tr6	Up7a	inside	T	Alpha helix	800
Tr7	Up	inside	T	extended	600
Tr8	Up7a	inside	T	extended	2000
Tr9	Up7a	outside	B	extended	1400
Tr10	Up	outside	B	extended	500

## Data Availability

The data presented in this study are available on request from the corresponding author.

## Supplementary Materials

The following supporting information can be downloaded at: https://www.mdpi.com/article/10.3390/membranes13040370/s1, Table S1: Thickness of the bilayer, area per lipid, rate of water permeability, an average number of water molecules inside the bilayer, Figure S1: The time evolution of the angle (in °) between the main principal axis of uperin peptide and the normal to the membrane surface for the trajectory Tr1.
