# Supplementary material for: Interaction of Uperin Peptides with Model Membranes: Molecular Dynamics Study
Source: Membranes (Basel). 2023 Mar 23;13(4):370. doi: 10.3390/membranes13040370 (PMC10146956; doi:10.3390/membranes13040370)
Supplement: Supplementary file 1 [file membranes-13-00370-s001.zip › membranes-2234950-supplementary.pdf]

membranes

MDPI

Supplementary Materials

Interaction of Uperin Peptides with Model Membranes: Molecular Dynamics Study

Elena A. Ermakova * and Rauf Kh. Kurbanov

Kazan Institute of Biochemistry and Biophysics, FRC Kazan Scientific Center of RAS, Lobachevsky Str. 2/31, Kazan 420111, Russia
* Correspondence: ermakova@kibb.knc.ru

Table S1. Thickness of the bilayer, area per lipid, rate of water permeability, an average number of water molecules inside the bilayer.

Trajectory Number	Thickness (Å)	Area per Lipid (Å^2)	Water/ Rate (ns^{-1})	Average Number of Water Molecules Inside the Bilayer
Tr1	41.4	59.8	0.018	1
Tr2	41.8	58.4	0.060	1
Tr3	42.7	58.4	0.030	2
Tr4	41.8	57.8	0.025	2
Tr5	41.8	59.5	0.207	12
Tr6	41.8	58.9	0.134	11
Tr7	41.2	59.5	0.052	5
Tr8	41.4	59.4	0.052	3
Tr9	39.8	62.9	0.240	1
Tr10	42.8	57.8	0.030	3
membrane	42.1	58.1	0.020	2

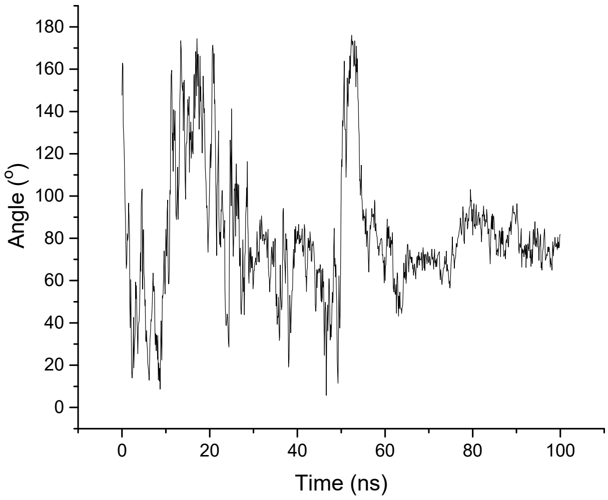

Figure S1. The time evolution of the angle (in °) between the main principal axis of uperin peptide and the normal to the membrane surface for the trajectory Tr1.
